# Novel Process Methods for the Whole Cottonseed: Effect on the Digestibility, Productivity, Fat Profile, and Milk Gossypol Levels in Lactating Dairy Cows

**DOI:** 10.3389/fnut.2022.801712

**Published:** 2022-02-15

**Authors:** Xiaoge Sun, Yitong Su, Yangyi Hao, Jun Zhang, Xiaomei Yue, Wei Wang, Zhu Ma, Kangkang Chu, Shuang Wang, Yajing Wang, Shengli Li

**Affiliations:** ^1^State Key Laboratory of Animal Nutrition, Beijing Engineering Technology Research Center of Raw Milk Quality and Safety Control, College of Animal Science and Technology, China Agricultural University, Beijing, China; ^2^College of Animal Science and Technology, Northwest A&F University, Yangling, China; ^3^Business Economics Group, Wageningen University & Research, Wageningen, Netherlands; ^4^Beijing Dairy Cattle Center, Beijing, China

**Keywords:** whole cottonseed, digestibility, processing methods, blood gossypol, fatty acid

## Abstract

In this study, we aimed to determine the effect of mixed-process methods on the ruminal degradability of whole cottonseed (WCS) both *in situ* and *in vitro*, and the effect on the production performance of dairy cows. Eight WCS process methods were tested on the ruminal digestibility, including crush-alkali 1 (CA1), crush-alkali 2 (CA2), crush-alkali 3 (CA3), alkali 1-crush (A1C), alkali 2-crush (A2C), alkali 3-crush (A3C), crush-only (CO), and non-processed. Alkali 1, 2, and 3 indicate the supplementation of alkali to WCS at the dose of 4% on dry matter (DM) base as followed: 4% NaOH, 2% NaOH + 2% CaO, and 2% NaOH + 2% CaCl_2_ alkaline, respectively. Among all treatments, CA2 showed the highest WCS ruminal degradation in situ and the highest intestinal digestibility of WCS *in vitro*. Furthermore, an animal experiment was conducted for 60 days on 30 Holstein dairy cows, using a diet without WCS (CON group), a diet containing 8% non-processed WCS (NP group), and a diet containing 8% CA2-treated WCS (CA2 group). The results indicated that the dry matter intake, 4% fat-corrected milk production, milk protein, milk fat, and content of short-chain saturated fatty acid of milk in the CA2 group were significantly higher (*P* < 0.05) than CON group. Furthermore, DMI, the CLA was significantly greater (*P* < 0.05) in the CA2 group than the other groups. Additionally, the free gossypol concentration in serum or milk was under safety level in the three groups. Overall, crush and alkalization (NaOH: CaO = 1:1) treatment could improve the utilization of WCS in dairy farms.

## Introduction

Whole cottonseed (WCS) is a by-product during the cotton ginning process, which can be used as an alternative feed ingredient for ruminants (1). The use of potentially human-inedible feed resources by livestock is very important for human food safety to meet the demands of a growing human population in the context of sustaining the production of high-quality foods from livestock.

WCS, characterized by high energy, moderately high crude protein, and highly effective fiber contents, is acknowledged to be a cost-effective premier feedstuff for lactating dairy cows (2). It has been found that compared with a control treatment, providing cows with a diet containing WCS increases the yields and the fat content of milk (3). Similarly, supplementary WCS in the feed elevates the fat, lactose, and protein contents in the milk and increases the proportion of long-chain fatty acid (LFA) and conjugated linoleic acid (CLA) by reducing the short- and medium-chain fatty acids (SFA, MFA) (4, 5). Moreover, supplementing the feed of lactating dairy cows with 2.61 kg/(head·day) WCS over a period of 12 weeks has been shown to result in a reduction in the magnitude of CH4 emissions from 13 to 26% (6), and has also been found to reduce the length of calving intervals and service period, and the number of days required for conception, as well as to promote the conception rate by 16% (7).

Despite of these beneficial effects, WCS supplementation is limited to a certain extent by some restrictive factors. For instance, gossypol was toxic to animals, and tannins and cottonseed hull could reduce the rumen digestibility of WCS (8). To alleviate the negative effects of WCS, different processing techniques have been designed to modify the natural structural substance and anti-nutritional factors of cottonseed, and subsequently, enhance the feeding efficiency of animals. Recent processing technologies have been directed toward non-thermal processing techniques due to their economic, environmental and sensorial benefits (9). Crushing and alkalization were two commonly used processing methods in by-products (10). It has been reported that crushed WCS could increase the feed efficiency of dairy cows (11). It also has been shown that the cottonseeds treated with alkali could increase availability of the nutrients by breaking the lignocellulose linkage (12). However, these studys as well as other earlier studies mainly focused on the effects of individually crushing processing (13, 14) or alkalization treatment on ruminants (15, 16). Moreover, the alkalization material used in those studys were NaOH, whereas the effect of WCS with CaO and CaCl_2_ treatment as well as the combination of crush and alkalization treatment on dairy cows were lacking. Therefore, it is meaningful to evaluate the synergetic effects of crushing associated with several different alkalization treatment (NaOH, CaO, CaCl2) from the perspectives of the digestibility characters of WCS *in situ* and *in vitro*, and the effect on the performance of lactating dairy cows *in vivo*.

In this study, we examined the utilization of an innovative mixed-processing method for WCS, namely, combined crushing and alkalization. The effects on ruminal degradability *in situ* and pepsin-pancreatin digestion *in vitro*, as well as animal performance, were investigated. Based on our findings, we had a new comprehensive assessment of multiple treatments for the efficient utilization of WCS on farms in China.

## Materials and Methods

### Processing of WCS by Crushing and Alkalization

The WCS, provided by the Beijing Zhongdi Shunyi Improved Cow Breeding Farm, had an impurity content of approximately 2% and dry matter content of 92.55% and was produced from Xinjiang Uygur Autonomous Region, China. The WCS was processed using the methods as followed (Table 1): crush-alkali treatment (CA); alkali-crush treatment (AC); crushing only (CO); non-processed WCS (NP). Crush-alkali treatment denotes initial crushing, followed by alkali treatment, whereas alkali-crush indicates alkali treatment prior to crushing. The amount of alkali added was 4% of the WCS (as DM basis). The WCS was crushed with a coarse-crusher machine (FW-400A, Hebi, China), and sieved to the particle size of 3–4 mm. The alkalization (A1, A2, and A3) was performed by adding three different mixed alkali materials at the ratio of 4% dry matter (DM) of WCS as follows: 4% NaOH (A1), 2% NaOH + 2% CaO (A2), and 2%NaOH + 2% CaCl_2_ (A3). Immediately after uniformly mixing the alkali solids (powder or small particles) with crushed WCS, water was slowly and evenly added while stirring at the ratio of 80% DM of WCS. The cottonseeds were soaked for 24 h, after which they were immediately transferred to the oven and dried at 65°C for 48 h.

**Table 1:** Crushing and alkalization treatments used to process WCS.

**Crushing and alkalization treatments^a^**	**The order of crushing and alkalization treatments^b^**	**Alkali composition (4% of the WCS DM weight)**
CA1	crush-alkali	NaOH
CA2	crush-alkali	NaOH:CaO = 1:1
CA3	crush-alkali	NaOH:CaCl_2_ = 1:1
A1C	alkali- crush	NaOH
A2C	alkali- crush	NaOH:CaO = 1:1
A3C	alkali- crush	NaOH:CaCl_2_ = 1:1
CO	crush	NA
NP	non-processing	NA

a *CA, crush-alkali treatment; AC, alkali-crush treatment; and A1, A2, and A3 are the mixed-alkali treatments, were 4% WCS DM using 4% NaOH, 2% NaOH + 2% CaO, and 2% NaOH + 2% CaCl_2_, respectively. CO, crushing only. NP, non-processed whole cottonseed (WCS)*.

b *Crush-alkali treatment denotes initial crushing, followed by alkali treatment, whereas alkali-crush indicates alkali treatment prior to crushing. The amount of alkali added was 4% of the WCS (as DM basis)*.

### Rumen Degradation *in situ*

Determinations of the rumen degradation rate of the major nutrients of processed cottonseed were based on the *in situ* method proposed by Orskov et al. (17). Three healthy high-yielding Holstein cows fitted with rumen fistulas were fed a total mixed ration (CP 16.9%) and milked three times daily for rumen degradation *in situ*. The ingredients of the diet included 12% alfalfa hay (DM basis), 4.5% oat hay, 30% corn silage, 15% soybean meal, 5% steam flak corn, 20% corn fine, 5% soybean hulls, 5% dried distillers grains and solubles, 0.5% sodium bicarbonate, 1% bypass fat product, and 2% premix. The dry matter intake (DMI) of each cattle was 24 kg/(cow·day), the net energy of lactation (NE_L_) of DM was (7.315 MJ/kg) and the total NE_L_ intake was 175.56 MJ/(cow·day). Cows were free to access diets and water.

We assessed rumen digestion of WCS after 2, 6, 12, 16, 24, 30, 36, 48, and 72 h of digestion. At each time point, 48 nylon bags (2 parallels × 8 treatment × 3 cows; pore size: 50 μm) containing approximately 10 to 15 g of WCS sample were weighed. A polyester mesh bag (32 × 45 cm with a 90 cm length of rope to be anchored to the cannula) was used to hold the nylon bags in the rumen. The nylon bags (2 parallels × 8 treatment) containing feed samples for each cow and each time point were placed in the rumen simultaneously and removed from rumen at the timepoint. After removal from the rumen, the bags were rinsed in cool running tap water until the wash water had become colorless. The washed bags were oven-dried at 65°C for 48 h and then weighed. The 0-h (two bags for each sample) incubation samples were not incubated in the rumen, but they were washed as described above. Residues from the bags were pooled within the incubation time and treatment. The residues in bags were determined for DM, CP, ether extract (EE), neutral detergent fiber (NDF), and acid detergent fiber (ADF) contents. The analytical DM content of the diet was determined by oven drying at 135°C for 2 h (18). NDF and ADF were determined using an ANKOM fiber analyzer (A2000i; American ANKOM, NY, USA), as described by Van Soest et al. (19). CP and EE were measured according to method 984.13 and 920.39 of the Association of Official Analytical Chemists (20), respectively. Net energy of lactation was calculated according to NRC (21).

### Small Intestine Digestion *in vitro*

To assess digestion in the small intestine, the residues of the different treated WCS after incubated in the rumen for 16 h were used for intestine digestion *in vitro*. Nylon bags with a pore size of 42–44 μm containing 0.5 g samples were treated with a hydrochloric acid solution (pH = 1) at 39°C for 2 h. After that, they were placed in a glass culture tube containing 1.0 g trypsin (SIGMA, P7545-500g, Mekemei Biomedical Technology Co., Ltd) and 2 g bile acid salt (BBI, BB0225-100 g, Shenggong Bioengineering Co., Ltd). The glass culture tube was then sealed and incubated at 39°C for 24 h in a constant temperature water bath. The trypsin and commercial bile salts were prepared in a McDougall buffer (pH 7.5) containing 98 g NaHCO_3_, 93 g Na_2_HPO_3_, 4.7 g NaCl, 1.2 g MgSO_4_·7H_2_O, 5.7 g KCl, and 0.8 g CaCl_2_·H_2_O, with a final volume of 10 L with distilled water.

After incubation *in vitro*, the nylon bags were rinsed with cold water until clean, dried at 65°C for 48 h, and weighed. The intestinal digestive residues of the WCS samples were collected in three replicates and were mixed for further analysis.

### Animal Feeding Experiment

#### Animals, Housing and Diets

The animal feeding experiment was carried out in Yanqing farm of the Beijing Dairy Cow Center from December 3, 2019, to February 12, 2020. Thirty healthy Holstein cows with similar daily milk production (29.20 ± 2.40 kg), parity (1.23 ± 0.43), and days of milk (99.03 ± 36.59) were selected and randomly divided into three groups (each containing 10 cows) as follows: Control (CON: with no supplementary WCS), non-processed WCS (NP: diet supplemented with 7.85% DM non-processed WCS), crush-alkali2 (CA2: diet supplemented with 7.85% CA2-treated WCS). The diet was formulated based on a concentrate to roughage ratio of 60:40 (Table 2). The CA2 was selected to take the feeding experiment, which was based on the former *in situ* and *in vitro* experiment results.

**Table 2:** Chemical composition and nutrient levels of the basal diet (% DM basis).

**Items**	**Groups**		
	**CON**	**NP**	**CA2**
**Ingredients**			
Corn silage	26.32	26.46	26.46
Spanish alfalfa hay	1.94	1.17	1.17
USA alfalfa hay	5.97	6.60	6.60
Domestic alfalfa hay	1.93	1.94	1.94
Domestic oat hay	6.09	3.16	3.16
Ground corn	17.80	14.97	14.97
Soybean meal (43%)	11.34	10.64	10.64
Extruded soybean	1.18	0.79	0.79
Canola meal	3.25	2.31	2.31
Steam-flaked corn	5.64	8.83	8.83
Spray corn bran	7.19	5.93	5.93
Soybean hulls	3.27	0.97	0.97
Whole cottonseeds	-	7.85	7.85
Fatty acid calcium	1.50	0.90	0.90
Fat powder	0.42	-	-
Beet pulp pellet	-	1.34	1.34
Yeast culture	1.94	1.95	1.95
Premix^a^	4.25	4.27	4.27
**Nutrient levels** ^b^			
NE_L_ (MJ/kg)	7.19	7.23	7.23
CP	16.80	17.12	16.98
NDF	30.21	31.04	30.89
ADF	16.50	17.93	17.76
EE	4.32	4.34	4.28
Starch	25.32	25.44	25.44
Free gossypol (mg/kg)	0	348	240

a *One kilogram of premix contained the following: Ca 139.11 g, P 0.01 g, Mg 52.86 g, K 73.01 g, S 1.09 g, Na 94.55 g, Cl 127.06 g, Fe 225.54 ppm, Mn 656.16 ppm, vitamin A 180.02KIU, vitamin D 30006.48 IU, vitamin E 550.03 IU*.

b *NE_L_, Net energy of lactation calculated according to NRC (21), while the other nutrient levels are measured values. CP, crude protein; NDF, neutral detergent fiber; ADF, acid detergent fiber; EE, ether extract*.

The experimental cattle were all maintained under free-stall conditions, fed *ad libitum*, and given free access to water. The cows were fed daily at 08:00 and 14:00, with residual feedstuff being <5%, and were milked three times daily (06:30, 13:30, and 20:30) in a 16 × 2 parallel milking parlor. DMI for each individual cow was recorded daily by the Roughage Intake Control (RIC) system (INSENTEC, Netherlands) consisting of 45 feeding banks and computer management software. Afigin milking management platform (Afimilk, Israel) was used to record daily milking production. The experiment was conducted over a 70-day period with the first 10 days as adaptation.

#### Milk Sample Collection and Analysis

During the experimental period, the milk yield was recorded daily, and the 4% FCM yield was calculated using model (1):


(1) $$\begin{array}{l} 4\%\text{FCM }=\;0.4\text{ M }+\;15\text{ F}, \end{array}$$

where 4% FCM denotes 4% fat-corrected milk (kg), M represents milk production (kg), and F represents the amount of milk fat (kg).

Samples were collected for analysis after 0, 15, 30, 45, and 60 days of feeding, with three replicate samples being collected for each cow from the flow diverters of the milking machine (9JP-2 ×16, DeLaVel, Sweden). Milk samples were collected in the morning, noon, and evening, and were mixed in a 4:3:3 ratio according to the proportion of the milk yield (approximately 4:3:3). These samples were divided into two 50-mL centrifuge tubes. To each tube, potassium dichromate preservative was added and stored at 4°C until further analysis. A second sample was sent to the Dairy Herd Improvement Center for analyses of milk composition using a near-infrared reflectance spectroscopy analyzer (Seris300 CombiFOSS; Foss Electric, Hillerød, Denmark), including milk fat (%), lactose (%), protein (%), and urea nitrogen (MUN) (mg/dL), along with a somatic cell count (SCC) (×1,000/mL). Methods currently used for quantitating fatty acid (FA) composition in milk requires solvent extraction, purification, and esterification followed by gas chromatographic (Agilent 6890, U.S.A) with a chromatographic column (DB-23, 60.0 m × 250 × 0.25 um) analysis described in Sukhija and Palmquist (22).

#### Blood Sample Collection and Analyses

Tail venous blood samples were collected from cows in 10-mL heparin tubes after 2 h morning feeding at 0, 15, 30, 45, and 60 days of the experimental period, and subsequently centrifuged for 10 min at 3,000 g. The serum thus obtained was divided into 2-mL freezing-storage tubes and stored at−20°C for subsequent analyses of biochemical indices, The following biochemical blood components were measured by an au-to-analyzer (CLS880, ZECEN Biotech Co., Ltd, China): globulin (GLB), total protein (TP), albumin (ALB), glucose (GLU) and triglyceride (TG), in which GLB, TP, and ALB were tested by kits from ZECEN Biotech Co., Ltd (Jiangsu, China), GLU and TG were tested by kits from Jiancheng Bioengineering Institute (Nanjing, China). β-hydroxybutyric acid (β-HBA) and nonesterified fatty acid (NEFA) levels were measured using a spectrophotometer (Model 722, Gaomi Caihong Analytical Instrument Co., China) with kits supported by Jiancheng Bioengineering Institute (Nanjing, China). Gossypol in blood samples was extracted according to the method described by Zhong (23). The contents of gossypol were quantified by high-performance liquid chromatography using a Wufeng analytical instrument (Wufeng Co., Ltd., Shanghai, China).

### Statistical Analysis

The ANOVA program of SAS 9.2 (SAS Institute Inc., Cary, NC, USA) and Duncan's method was used to analyze the effects of different processing methods on the effective ruminal degradation, intestinal digestibility, and free gossypol content of WCS, and multiple comparisons between the groups were performed based on the following model (2):


(2) $$\begin{array}{l} \text{Yij }=\;\mu \;+\text{ treati }+\text{ eij}, \end{array}$$

where Yij represents the effective ruminal degradation, small intestinal digestibility, or free gossypol content of the main components of processed cottonseed, μ represents the overall average, treati represents different treatment methods, and eij represents random residuals. Data analysis results are expressed as the means ± standard deviation (SD). Differences were considered significant at the *P* < 0.05 level, whereas 0.05 ≤ *P* ≤ 0.1 was taken to be indicative of a different trend.

The original SCC data were initially log converted to conform to the normal distribution, prior to being used for subsequent statistical analysis. The milk composition and blood index values were analyzed using SAS 9.2 ANOVA and multiple comparisons were performed using the Duncan test. Covariance analysis of milk production was performed using the SAS 9.2 GLM program and model (3), as follows:


(3) $$\begin{array}{l} \text{MYijk }=\;\mu \;+\text{ FEEDi }+\;\beta \text{Xj }+\text{ eijk}, \end{array}$$

where MYijk is milk yield (kg/d), μ represents the population means, FEEDi is the diets of different experimental groups (CON, NP, CA2), Xj is the DIM of the cow (days), β is a regression coefficient of milk yield and covariate DIM, and EIJK was a random residual.

The dephenolization efficiency was calculated using model (4):


(4) $$\begin{array}{l} \text{Dephenolization efficiency }=\;100\;\times \;(\text{free gossypol content of}\\ \text{WCS before processing }-\text{ free gossypol content after}\\ \text{processing})/\text{free gossypol content before processing}, \end{array}$$

Data analysis results are expressed as the means ± standard deviation (SD). Differences were considered significant at the *P* < 0.05 level, whereas 0.05 ≤ *P* ≤ 0.1 was taken as a tendency difference.

## Results

### Effects of Different Processing Methods on the Nutritents and Free Gossypol Content of WCS

**Figure 1:**
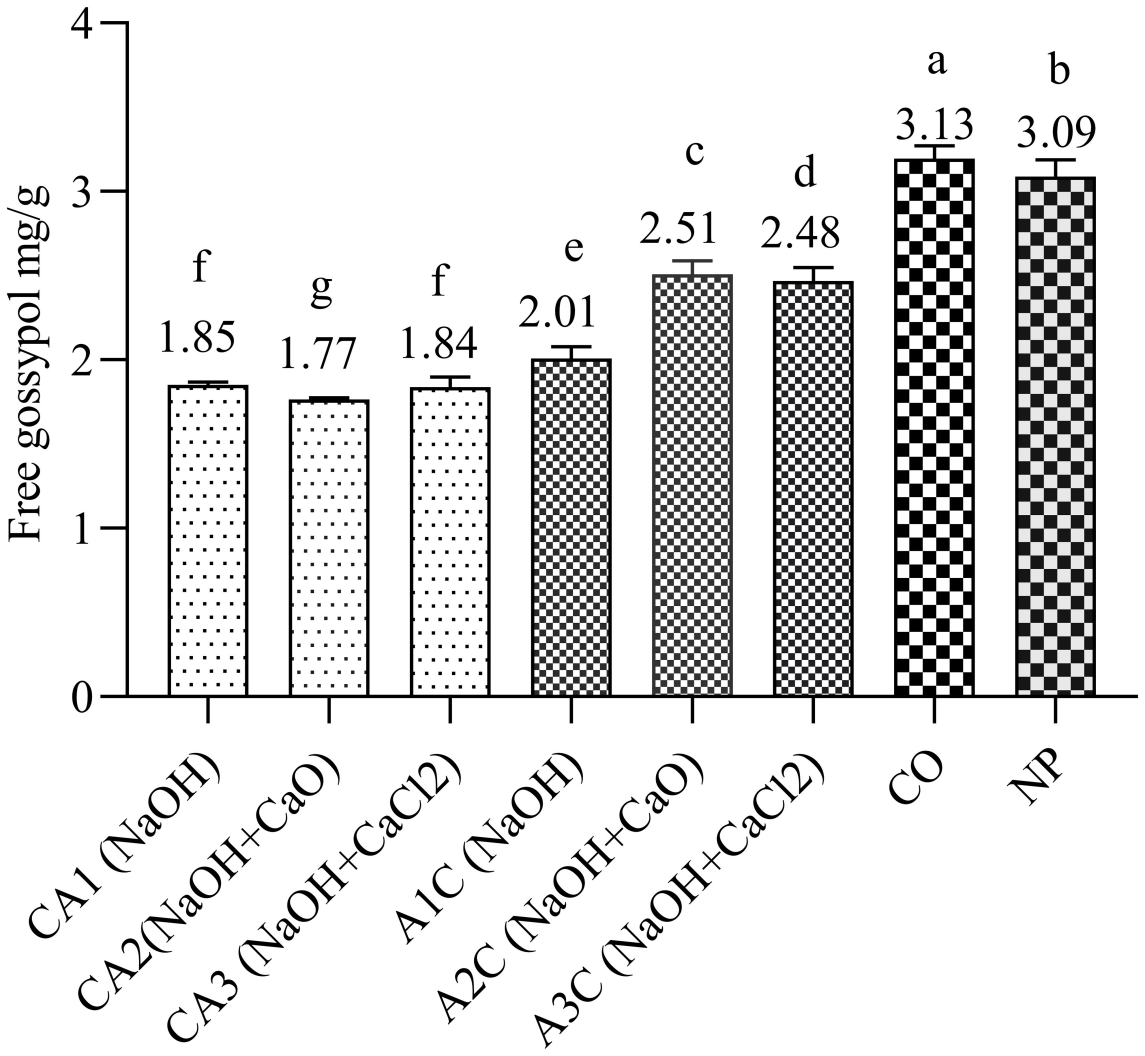
The effect of different processing methods on free gossypol content of WCS. In the column diagram above, values with different small letter superscripts indicate significant difference (*P* < 0.05), while with the same letter superscripts indicate no significant difference (*P* > 0.05).

Analyses revealed that the CP, EE, NDF, and ADF contents of WCS processed among groups (CA, AC, and CO) were all similar (*P* > 0.05) with those in unprocessed WCS (NP group) (Supplementary Table 1). The free gossypol content was significantly lower in CA groups than AC groups (*P* < 0.05), whilst CA2-processed WCS had the lowest among all treatments (Figure 1). Moreover, compared with the NP group, the dephenolization efficiency of the CA2 treatment (NaOH: CaO = 1:1, 4% mixed alkali solid solution method) was approximately 42.58%.

### Effects of Different Processing Methods on the Ruminal Degradation of WCS *in situ*

The effective ruminal degradation rates of the DM, CP, EE, NDF, and ADF contents of WCS in the NP group were significantly lower (*P* < 0.05) than those in the other groups (Table 3). The effective ruminal degradation rates of CP and EE in the CA groups (i.e., CA1, CA2, CA3) were significantly lower (*P* < 0.05) than those in the AC groups (i.e., AC1, AC2, AC3) and CO group. Among the different processing treatments, the CA2 treatment was found to have the most pronounced rumen-protective effect with respect to EE (Table 3), while this treatment was observed to be the most conducive to enhancing the ruminal degradation of NDF and ADF (Table 3).

**Table 3:** Effect of different alkali mixtures on the coefficient of ruminal degradation of WCS *in situ*.

**Alkali mixtures**	**DM**	**CP**	**EE**	**NDF**	**ADF**
CA1	0.420 ± 0.025^b^	0.583 ± 0.050^c^	0.604 ± 0.023^c^	0.373 ± 0.025^b^	0.320 ± 0.023^b^
CA2	0.466 ± 0.048^a^	0.592 ± 0.054^c^	0.568 ± 0.029^c^	0.452 ± 0.031^a^	0.420 ± 0.026^a^
CA3	0.419 ± 0.024^b^	0.556 ± 0.028^c^	0.570 ± 0.097^c^	0.437 ± 0.036^a^	0.404 ± 0.037^a^
A1C	0.459 ± 0.070^ab^	0.631 ± 0.019^b^	0.807 ± 0.095^b^	0.405 ± 0.091^b^	0.336 ± 0.086^b^
A2C	0.445 ± 0.070^ab^	0.653 ± 0.018^b^	0.778 ± 0.052^b^	0.386 ± 0.063^b^	0.293 ± 0.029^bc^
A3C	0.426 ± 0.074^b^	0.656 ± 0.031^b^	0.806 ± 0.017^b^	0.365± 0.080^b^	0.313 ± 0.052^bc^
CO group	0.492 ± 0.022^a^	0.728 ± 0.016^a^	0.829 ± 0.018^a^	0.318 ± 0.065^c^	0.281 ± 0.011^c^
NP group	0.300 ± 0.027^c^	0.408 ± 0.024^d^	0.434 ± 0.011^d^	0.204 ± 0.015^d^	0.195 ± 0.022^d^

*Alkali mixtures, refer to Table 1 alkali mixtures methods. DM, dry matter; CP: crude protein; EE, ether extract; NDF, neutral detergent fiber; ADF, acid detergent fiber. Values in the same column denoted by different superscript lowercase letters indicate significant differences (P < 0.05), whereas those denoted by the same letters (P > 0.05)*.

### Effects of Different Processing Methods on the Intestinal Digestibility of WCS *in vitro*

As shown in Table 4, the intestinal digestibility of WCS in the CA groups was significantly higher than that of the AC and CO groups (*P* < 0.05). Among the three CA groups, we found that the highest intestinal digestibility of the DM and EE was achieved in treatment CA2 was highest (*P* < 0.05) (Table 4). The intestinal digestibility of DM, CP, and EE of CA2-processed WCS was also significantly higher (*P* < 0.05) than in other treatment groups (Table 4).

**Table 4:** The effect of different alkali mixtures on the coefficient of intestinal digestibility of WCS *in vitro*.

**Alkali mixtures**	**DM**	**CP**	**EE**
CA1	0.347 ± 0.039^b^	0.588 ± 0.040^b^	0.369 ± 0.061^c^
CA2	0.394 ± 0.026^a^	0.618 ± 0.092^a^	0.580 ± 0.038^a^
CA3	0.329 ± 0.036^b^	0.531 ± 0.086^c^	0.408 ± 0.083^b^
AC1	0.304 ± 0.015^c^	0.527 ± 0.083^c^	0.393 ± 0.040^bc^
AC2	0.302 ± 0.024^c^	0.530 ± 0.064^c^	0.463 ± 0.083^b^
AC3	0.287 ± 0.019^c^	0.514 ± 0.089^c^	0.385 ± 0.064^c^
CO group	0.308 ± 0.08^c^	0.550 ± 0.061^bc^	0.443 ± 0.030^b^
NP group	0.364 ± 0.017^ab^	0.588 ± 0.024^b^	0.452 ± 0.035^b^

*Alkali mixtures, refer to Table 1 alkali mixtures methods. DM, dry matter; CP, crude protein; EE, ether extract; NDF, neutral detergent fiber; ADF, acid detergent fiber. Values in the same column denoted by different superscript lowercase letters indicate significant differences (P < 0.05), whereas those denoted by the same letters are not significantly different (P > 0.05)*.

### Effects of Processed WCS on Blood Parameters, DMI, Lactation Performance, and Content of Free Gossypol in the Milk of Dairy Cows

The DMI of CA2 group cows was significantly higher than other groups (*P* < 0.05), whereas no statistically significant difference in DMI was detected between the CON and NP groups (Table 5). Cows fed on a diet supplemented with CA2-processed WCS were observed to have a higher 4% FCM yield (*P* < 0.05), which was 3.7 kg/(head/day) higher as compared with CON group cows. Furthermore, the fat and protein contents of milk in NP and CA2 groups were significantly higher than that of the CON group (*P* < 0.05), with milk fat being 7.73 and 11.60% higher, respectively (Table 5). In contrast, there were no significant differences found among the three groups with respect to milk lactose, MUN, and SCC (Table 5). Finally, the free gossypol content of milk did not differ between different treatments (*P* > 0.05) (Table 5).

**Table 5:** Effects of processed WCS on dry matter intake, milking performance, and milk composition.

**Items**	**Groups**		
	**CON**	**NP**	**CA2**
DMI [kg/(head·day)]	19.98 ± 1.60^b^	19.08 ± 2.79^b^	22.81 ± 1.58^a^
**Milking performance**			
Milk Yield [kg/(head·day)]	26.69 ± 0.82	27.51 ± 0.82	28.45 ± 0.86
4%FCM [kg/(head·day)]	26.15 ± 0.87^b^	28.25 ± 0.87^ab^	29.85 ± 0.92^a^
**Milk composition**			
Fat percentage (%)	3.88 ± 0.42^b^	4.18 ± 0.54^a^	4.33 ± 0.59^a^
Protein percentage (%)	3.26 ± 0.18^b^	3.44 ± 0.28^a^	3.49 ± 0.33^a^
Lactose percentage (%)	5.26 ± 0.23	5.28 ± 0.14	5.34 ± 0.19
MUN (mg/dL)	13.04 ± 2.78	12.92 ± 3.48	13.42 ± 2.94
SCC (×1,000/mL)	57.54 ± 2.57	48.98 ± 2.57	59.21 ± 3.16
**Food safety**			
Free gossypol (mg/L)	0.00 ± 0.00	0.11 ± 0.05	0.12 ± 0.04

*CON, diet without whole cottonseed (WCS); NP, diet added with 8% unprocessed WCS; CA2, diet supplemented with 8% crush-alikali2-treated WCS. Values in the same row denoted by different superscript lowercase letters indicate significant differences (P < 0.05), whereas those denoted by the same letters or no letter are not significantly different (P > 0.05). MUN, Milk urea nitrogen. SCC, Somatic cell count*.

As shown in Table 6, there were no significant differences in blood parameters reflecting liver function or energy metabolism among the three groups. Meanwhile, the serum gossypol content was not affected by CA2 treatment compared with NP treatment.

**Table 6:** Effects of processed WCS on serum biochemical parameters and free gossypol content.

**Items**	**Groups**		
	**CON (*n* = 10)**	**NP (*n* = 10)**	**CA2 (*n* = 10)**
**Liver function**			
ALB (g/L)^a^	38.54 ± 1.27	38.69 ± 1.75	38.54 ± 1.62
GLB (g/L)	36.30 ± 3.50	35.64 ± 4.00	34.73 ± 3.32
ALB/GLB	1.07 ± 0.10	1.10 ± 0.13	1.12 ± 0.12
TP (g/L)	74.84 ± 4.05	74.33 ± 3.99	73.27 ± 3.56
**Energy metabolism**			
GLU (mmol/L)	2.80 ± 1.02	3.28 ± 0.74	2.74 ± 1.03
TG (mmol/L)	0.29 ± 0.04	0.28 ± 0.04	0.28 ± 0.04
NEFA (μmol/L)	51.28 ± 7.07	51.03 ± 9.84	49.55 ± 8.68
β-HBA (mmol/L)	0.43 ± 0.05	0.46 ± 0.09	0.43 ± 0.08
**Toxin indicator**			
Serum free gossypol (mg/kg)	0.00 ± 0.00	0.18 ± 0.04	0.15 ± 0.07

*GLU, glucose, ALB, albumin; GLB, globulin; TP, total protein; TG, triglyceride; β-HBA, β-hydroxybutyric; NEFA, non-esterified fatty acid. CON, diet without whole cottonseed (WCS); NP, diet added with 8% unprocessed WCS; CA2, diet supplemented with 8% crush-alkali 2 treated WCS. Values in the same row denoted by different superscript lowercase letters indicate significant differences (P < 0.05), whereas those denoted by the same letters or no letter are not significantly different (P > 0.05)*.

### Effects of Processing WCS on the Milk FA Composition

**Figure 2:**
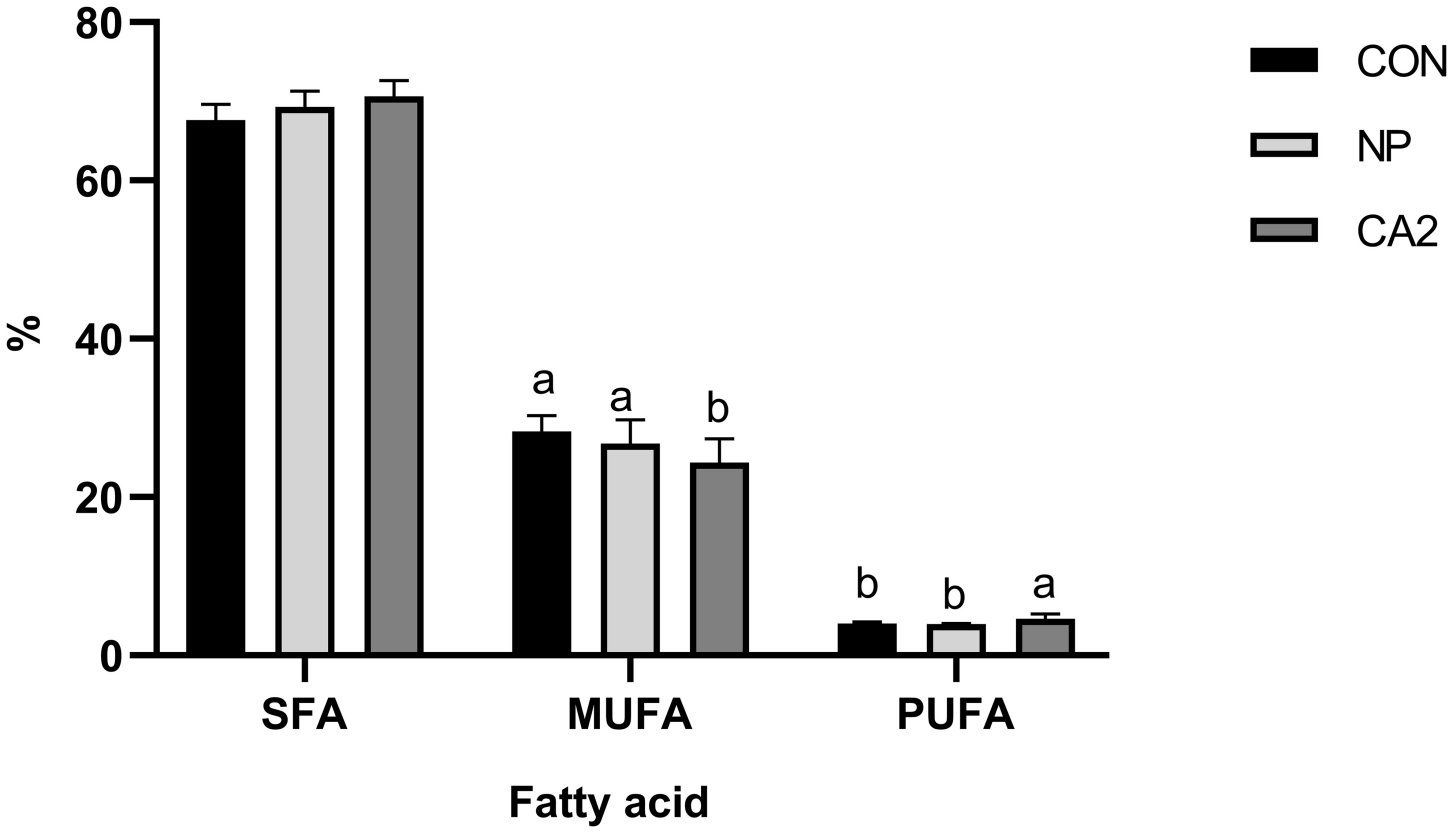
Effects of processed whole cottonseed (WCS) on the milk fat profile. SFA, saturated fat acid; MUFA, monounsaturated fatty acid; PUFA, polyunsaturated fatty acids. CON, diet without WCS; NP, diet added with 8% unprocessed WCS; CA2, diet supplemented with 8% crush-alikali2-treated WCS. In the column diagram above, values with different small letter superscripts indicate significant difference (*P* < 0.05), while with the same or no letter superscripts indicate no significant difference (*P* > 0.05).

In each of the treatment groups, the FA profiles of milk mainly contained MFA and SFA, whereas the majority of LFA was identified as C18 FA (Table 7). Compared with the CON group, the contents of C6:0, C8:0, C10:0, C12:0, C13:0, and C14:0 SFA were significantly increased by CA2 supplementation (*P* < 0.05). CA2 also promoted the levels of CLA in milk, which was approximately 6.67% higher than the other two groups (*P* < 0.05), and tans-9, cis-11 CLA higher than the CON group (*P* < 0.05). The omega (ω)-6 FA and the ω-6/ω-3 ratio was significantly higher in the CA2 group than the other two groups (*P* < 0.05). Compared with the CON group, SFA was significantly elevated in the milk of CA2 group cows (*P* < 0.05), whereas there were no significant differences between the NP and CA2 group cows with respect to MFA and LFA (*P* > 0.05) (Table 7). Moreover, compared with the NP and CON groups, the CA2 group showed significantly lower levels of monounsaturated fatty acid (MUFA), but 26% higher levels of polyunsaturated fatty acid (PUFA). Even though, all groups showed similar saturated FA in the milk (*P* > 0.05) (Figure 2).

**Table 7:** Effects of processed WCS on milk fatty acid composition (%).

**Items**	**Groups**		
	**CON**	**NP**	**CA2**
C6:0^3^	1.33 ± 0.11^b^	1.42 ± 0.10^ab^	1.45 ± 0.10^a^
C8:0	1.08 ± 0.10^b^	1.16 ± 0.09^ab^	1.20 ± 0.11^a^
C10:0	2.44 ± 0.29^b^	2.78 ± 0.36^a^	3.03 ± 0.40^a^
C12:0	3.07 ± 0.41^b^	3.46 ± 0.48^ab^	3.84 ± 0.58^a^
C13:0	0.12 ± 0.02^b^	0.15 ± 0.04^ab^	0.16 ± 0.03^a^
C14:0	10.74 ± 0.80^b^	11.12 ± 0.79^ab^	11.71 ± 0.85^a^
C14:1	0.92 ± 0.18	0.79 ± 0.23	0.78 ± 0.17
C15:0	1.04 ± 0.12	1.11 ± 0.18	1.18 ± 0.11
C16:0	35.00 ± 1.67	33.82 ± 2.02	33.78 ± 1.24
C16:1	1.37 ± 0.11^a^	1.31 ± 0.24^ab^	1.15 ± 0.16^b^
C17:0	0.56 ± 0.03	0.57 ± 0.02	0.58 ± 0.04
C18:0	11.79 ± 1.29	13.23 ± 1.71	13.16 ± 1.47
C18:1n9c	25.56 ± 1.80^a^	24.24 ± 2.16^a^	22.05 ± 2.55^b^
C18:2n6c	2.98 ± 0.36^b^	2.89 ± 0.36^b^	3.87 ± 0.39^a^
C18:3n3	0.34 ± 0.04	0.30 ± 0.04	0.31 ± 0.03
CLA-c9t11	0.23 ± 0.04^b^	0.24 ± 0.04^ab^	0.26 ± 0.05^a^
CLA-t10c12	0.05 ± 0.01	0.05 ± 0.01	0.05 ± 0.01
C20:0	0.20 ± 0.02	0.21 ± 0.03	0.21 ± 0.03
C20:1	0.12 ± 0.02	0.11 ± 0.01	0.11 ± 0.02
C20:2	0.06 ± 0.01	0.07 ± 0.02	0.08 ± 0.02
C20:3n6	0.05 ± 0.01	0.06 ± 0.02	0.06 ± 0.02
C20:4n6	0.17 ± 0.02	0.16 ± 0.03	0.18 ± 0.03
C20:5n3	0.19 ± 0.03	0.30 ± 0.03	0.22 ± 0.03
C21:0	0.10 ± 0.00	0.10 ± 0.01	0.09 ± 0.00
C22:0	0.03 ± 0.00	0.03 ± 0.00	0.03 ± 0.00
C22:1n9	0.32 ± 0.04	0.29 ± 0.04	0.29 ± 0.03
C23:0	0.07 ± 0.00	0.08 ± 0.01	0.09 ± 0.06
C24:0	0.05 ± 0.01	0.05 ± 0.01	0.05 ± 0.00
C24:1	0.02 ± 0.00	0.02 ± 0.00	0.02 ± 0.00
CLA	0.29 ± 0.01^b^	0.30 ± 0.01^b^	0.32 ± 0.01^a^
ω-6 PUFA	3.46 ± 0.41^b^	3.38 ± 0.42^b^	4.40 ± 0.38^a^
ω-3PUFA	0.37 ± 0.04	0.33 ± 0.04	0.34 ± 0.03
ω-6 / ω-3	9.43 ± 0.60^c^	10.20 ± 0.30^b^	12.98 ± 0.74^a^
SFA (<C14)^3^	8.04 ± 0.84^b^	9.96 ± 1.02^ab^	9.69 ± 1.12^a^
MFA (C14 C16)	49.07 ± 2.25	48.15 ± 2.98	48.61 ± 2.02
LFA (>C16)	42.90 ± 2.62	42.89 ± 3.68	41.70 ± 2.79

*Different types of fatty acids. CLA, conjugated linoleic acid; PUFA, polyunsaturated fatty acid; MUFA, monounsaturated fatty acid; SFA, short-chain saturated fatty acid; MFA, medium-chain saturated fatty acid; LFA, long-chain saturated fatty acid. CON, diet without whole cottonseed (WCS); NP, diet added with 8% unprocessed WCS; CA2, diet supplemented with 8% crush-alikali2-treated WCS. ^3^In the same row, values with different small letter superscripts mean significant difference (P < 0.05), while with the same or no letter superscripts mean no significant difference (P > 0.05)*.

## Discussion

The hard husk of unbroken or non-chewed WCS can inhibit the degradation of cottonseed by rumen microorganisms. For the traditional cultivars of cotton, the rumen degradation rate of WCS *in situ* was only 12.8%, which can reach up to 58.6% after pre-crushing (24). In the current study, We found that the combination of crushing and alkalization process considerably enhanced rumen DM digestibility coefficient, with a range between 41.9 and 49.2%, while the DM digestibility coefficient of non-processed WCS was only 30.0%. These results may due to the fact that crushing contributes to the release of nutrients in the WCS and increases the area of contact between the rumen microbiota and cottonseed. On the other hand, alkalization is conducive to breaking the links between hemicellulose and lignin in cottonseed conchiolin and swells cottonseed hulls. The cotton kernel and hull NDF polysaccharides are generally more accessible to rumen microorganisms, and the alkaline hydrolysis of cellulose enhances the degradation of WCS nutrients (25). It supported our study that the WCS treated with the combination method of crush and alkalization could increase the rumen digestibility of NDF and ADF when compared with only crushed treatment and non-processed WCS.

In our study, the alkalinization method inhibited the ruminal digestibility of cottonseed CP, since the degradation of WCS CP following combined crushing and alkalization treatment was lower than that of WCS singularly processed by crushing. These negative effects of alkalinization on ruminal digestibility are consistent with the findings of (16) and (26). It may due to the protein were bound to other chemical componets by alkali (27, 28). However, the ruminal degradation of crushing cottonseed CP was enhanced compared with unprocessed WCS in our study. It indicated that the positive effects of crushing on the degradation of cottonseed overweighted the inhibitory effect of alkalization on CP rumen degradation. Our study revealed that the CP rumen degradation in AC groups was higher than in CA groups, which may be because the crushing prior to alkali treatment enlarged the alkali contact area. Likewise, we found that the overall rumen protective effect of CA groups on EE was superior to that of the AC groups. Meanwhile, the CA2 treatment had the greatest protection of EE in rumen degradation. This can be explained by Gadeyne et al. (29) that calcium salts *per se* tended to be resistant to degradation by ruminal microorganisms and could protect FA from bacterial digestion, and crush prior to alkali enlarge the protected area of CP. This would thus enable us to gain a better understanding of the protective effect of cottonseed protein and fat in the rumen.

In the present study, compared with AC treatments and CO treatment, most of the CA treatments had higher *in vitro* intestinal digestibility of WCS, DM, and CP, and CA2 treatment had the highest *in vitro* intestinal digestibility. It could be partially explained that alkalization was beneficial for the intestinal digestibility of cottonseed DM and CP, and the CA groups had much larger alkali contact with WCS (16). In summary, the CA2 method was the most effective process regarding the enhancement in the utilization of WCS main nutrients (i.e., DM, NDF, ADF, CP, and EE) *in situ* rumen degradation and *in vitro* intestinal digestion.

In the present study, we found that CA2 processing methods significantly improved the DMI of dairy cows. This result was in line with previous studies that showed alkalized cottonseed increased 2 kg DMI (25), promoted the ruminal degradation of NDF and ADF, and enhanced the feeding frequency and nutrient intake of cows (30) when compared with unprocessed WCS. It also coincided with the results in our study that WCS nutrients *in situ* rumen degradation and *in vitro* intestinal digestion were increased. Additionally, our results suggested that the higher NDF digestibility may result in the higher milk fat and 4% FCM of the CA2 group due to the improvement of FA biosynthesis in milk and milk production, which was consistent with the study of Solomon, Adin, Mabjeesh, Nikbachat, Yosef, Ben-Ghedalia and Miron (25). For the milk fat composition, we found the CA2 group had a higher level content of C18:2 FA than the other two groups, which could be explained by the consequence of a higher rate of ruminal fat protection of CA2 treated WCS compared with non-processed WCS, thereby decreasing the efficiency of biohydrogenation in the rumen and increasing the proportion of C18:2 leaving the rumen (31). CLA, a derived species of long-chain fatty acids, occurs in several isomeric forms mainly in cis-9, trans-11CLA, and trans10, cis12 CLA. CLA has many biological functions and multiple beneficial effects concerning anticancer activity, immunity enhancement, and regulation of lipid metabolism (32). In the present study, we observed that CLA content in CA2 group cows was higher than that other two treatment groups. It could be explained by CA2 treated WCS increased the C18:2 and C18:3 FA in rumen which may serve as precursors for cis-9,trans-11 CLA by partial ruminal biohydrogenation (33). According to the study of (34), CLA could cause substantial reductions in milk fat percentage, which has been termed the “low-fat milk syndrome.” However, in our study, we did not found the “low-fat milk syndrome” phenomenon in the CA2 group, even though associated with higher CLA in milk. It could be partially explained by the higher NDF as mentioned before. Another resonse may because the trans-10, cis-12 CLA was thought to play a key role in milk fat depression (35), and the trans-10, cis-12 CLA content in milk was not changed among the three treatments. The higher CLA in the CA2 group was mainly caused by the higher cis-9, trans-11 CLA. Previous studies indicated that cis-9, trans-11 CLA had multiple health benefits such as reduction of body fat mass, anti-atherogenic, anticarcinogenic, and immune-modulating effects (36, 37). Therefore, supplementation of WCS to the diet of dairy cows could act as an effective strategy to increase the fat content in the milk of dairy cows.

Ruminants with a well-developed ruminal microbial population were able to detoxify gossypol by converting the free gossypol to bound gossypol within the rumen, thereby inhibting its absorption into the blood (38). Generally, diets supplemented with 10–15% WCS did not contribute to the production of excessive amounts of free gossypol in either blood or milk (39). However, it was possible that feeding excessive amounts of gossypol in the free form may exceed this protective mechanism and impair animal performance. It has been shown that feeding large amounts of cottonseed was the possibility of gossypol toxicity and a potential depression in fertility in dairy cows (40). The U.S. Food and Drug Administration has established a tolerance level of 0.045% for free gossypol in cottonseed meals (41). In the present study, we found that cows fed a diet supplemented with 8%WCS showed no evident clinical symptoms of gossypol poisoning, and the levels of free gossypol in the milk produced by each group of cows were < 0.12 mg/kg, which is considerably much lower than the accepted standard, there-by indicating that the WCS would not cause food safety and animal health problems.

In this study, we found no significant differences in MUN and SCC among treatments, and the recorded values were all within an appropriate range. Moreover, during the trial period, the cattle in all treatment groups retained good health. It implied that the addition of WCS would not affect the health of lactation cows. In the present study, there were no significant differences in the serum contents among the treatment groups. The serum contents of NEFA, β-HBA and TG reflect the energy metabolism, while TP, GLB, and ALB reflect the liver function. The blood parameters reflecting liver function were not shifted, which may be because gossypol did not affect the liver function due to the detoxification by ruminants through the binding of soluble proteins to gossypol (42). Elevated blood lipids have been observed with increased absorption of supplemental dietary fat (43). However, the intake of EE did not vary in the present study, and a change in plasma NEFA, β-HBA, and TG concentration would not be expected unless cows were in different states of adipose tissue mobilization (31). Therefore, the results indicated that the CA2 process did not have any pronounced detrimental effect on the energy metabolism and liver function of dairy cows.

In conclusion, the CA2 treatment significantly increased the DMI of dairy cows, the yield of 4% FCM, milk protein percentage, milk fat percentage, and CLA in milk, which may mainly be due to the increase of the effective rumen degradation of DM, NDF, ADF, the greater rumen protection of EE, and the greater small intestinal digestibility of DM, CP, and EE. A diet supplemented with 8% (DM basis) CA2-processed cottonseed does not impair liver function or disrupt normal energy metabolism, and would not contribute to the cumulative toxicity of free gossypol in serum. Moreover, the higher CLA produced in milk would enhance the beneficial properties of milk, and thus human health. It suggested that the crush and alkalization (NaOH: CaO = 1:1) treatment prior to feeding animals could maximize the utilization of WCS in a dairy farm, but the biggest addition amount still needed to be studied in the future.

## Data Availability Statement

The original contributions presented in the study are included in the article/Supplementary Material, further inquiries can be directed to the corresponding author.

## Ethics Statement

All experimental procedures were approved by the Ethical Committee of the College of Animal Science and Technology of China Agriculture University (Protocol number: CAU20190924-1).

## Author Contributions

SL and JZ: conceptualization. YW: methodology. YS: software and validation. XS: formal analysis, writing—original draft preparation, and visualization. XS, YS, YH, KC, SW, and ZM: investigation. JZ and XY: resources. YH: data curation. XY: writing—review and editing. SL: supervision and funding acquisition. WW: project administration. All authors have read and agreed to the published version of the manuscript.

## Funding

This work was supported by the Major S&T projects of Xinjiang Uygur Autonomous Region (2020A01001) and the 2115 Talent Development Program of China Agricultural University.

## Conflict of Interest

The authors declare that the research was conducted in the absence of any commercial or financial relationships that could be construed as a potential conflict of interest.

## Publisher's Note

All claims expressed in this article are solely those of the authors and do not necessarily represent those of their affiliated organizations, or those of the publisher, the editors and the reviewers. Any product that may be evaluated in this article, or claim that may be made by its manufacturer, is not guaranteed or endorsed by the publisher.
