# Supplementary material for: Novel Process Methods for the Whole Cottonseed: Effect on the Digestibility, Productivity, Fat Profile, and Milk Gossypol Levels in Lactating Dairy Cows
Source: Front Nutr. 2022 Feb 15;9:801712. doi: 10.3389/fnut.2022.801712 (PMC8886631; doi:10.3389/fnut.2022.801712)
Supplement: Supplementary file 1 [file Table_1.DOCX]

Supplementary Material

Novel process methods for the whole cottonseed: Effect on the digestibility, productivity, fat profile, and milk gossypol levels in lactating dairy cows

**Xiaoge Sun ^1^, Yitong Su^1✝^, Yangyi Hao^1^, Jun Zhang^2^, Xiaomei Yue^3^, Wei Wang^1^, Zhu Ma^4^, Kangkang Chu^4^, Shuang Wang^4^, Yajing Wang^1^, Shengli Li^1*^**

^1^ State Key Laboratory of Animal Nutrition, Beijing Engineering Technology Research Center of Raw Milk Quality and Safety Control, College of Animal Science and Technology, China Agricultural University, No. 2 Yuanmingyuan West Road, Haidian, Beijing 100193, P. R. China

^2^ College of Animal Science and Technology, Northwest A&F University, Yangling 712100, Shaanxi, China, E-mail: jzhang0701@nwafu.edu.cn

^3^ Business Economics Group, Wageningen University & Research, 6706 KN Wageningen, the Netherlands

^4^ Beijing Dairy Cattle Center (BDCC), Beijing, P.R. China

✝ Co-author

*** Correspondence:**

Shengli Li, E-mail: lisheng0677@163.com; Tel: 86+13331168629; Fax: 00+86+62731254

Table S1. The nutrient contents of different processed whole cottonseed (WCS)

| Processing methods^1^ | Nutrient content of WCS (DM basis) % | | | | |
| --- | --- | --- | --- | --- | --- |
|  | DM | CP | EE | NDF | ADF |
| CA1 (NaOH) | 92.18±0.70 | 21.50±1.16 | 16.68±1.55 | 44.72±3.70 | 34.86±2.59 |
| CA2 (NaOH+CaO) | 92.06±0.62 | 20.92±1.12 | 15.98±1.61 | 46.96±3.67 | 34.59±2.48 |
| CA3 (NaOH+CaCl_2_) | 92.69±0.72 | 19.79±1.11 | 14.44±1.49 | 43.63±3.62 | 32.23±2.36 |
| A1C (NaOH) | 92.54±0.79 | 21.84±1.17 | 14.94±1.56 | 42.10±3.77 | 31.42±2.44 |
| A2C (NaOH+CaO) | 93.03±0.80 | 20.59±1.05 | 15.95±1.60 | 40.58±3.61 | 29.90±2.49 |
| A3C (NaOH+CaCl_2_) | 93.15±0.81 | 20.38±1.15 | 14.51±1.57 | 39.89±3.54 | 30.83±2.56 |
| CO | 91.02±0.82 | 21.81±1.23 | 16.58±1.61 | 51.54±3.76 | 41.04±2.67 |
| NP | 91.42±0.70 | 23.41±1.33 | 19.35±1.61 | 48.30±3.88 | 36.46±2.33 |

^1^CA: crush-alkali treatment, AC: alkali-crush treatment; and A1, A2, and A3 are the mixed-alkali treatments, were 4% WCS dry matter (DM) using 4% NaOH, 2% NaOH + 2% CaO, and 2% NaOH + 2% CaCl_2_, respectively. CO: crushing only. NP: non-processed whole cottonseed (WCS). Crush-alkali treatment denotes initial crushing, followed by alkali treatment, whereas alkali-crush indicates alkali treatment prior to crushing. The amount of alkali added was 4% of the WCS (as DM basis).

^2^Values in the same row denoted no letter mean there was no significant difference (*P* > 0.05).
